# Supplementary material for: GLYCO-2: a Tool to Quantify Glycan Shielding of Glycosylated Proteins with Improved Data Processing and Computation Speed
Source: bioRxiv. 2025 Feb 27:2025.02.26.640297. Preprint. [Version 1] doi: 10.1101/2025.02.26.640297 (PMC12190171; doi:10.1101/2025.02.26.640297)
Supplement: Supplement 1 [file media-1.pdf]

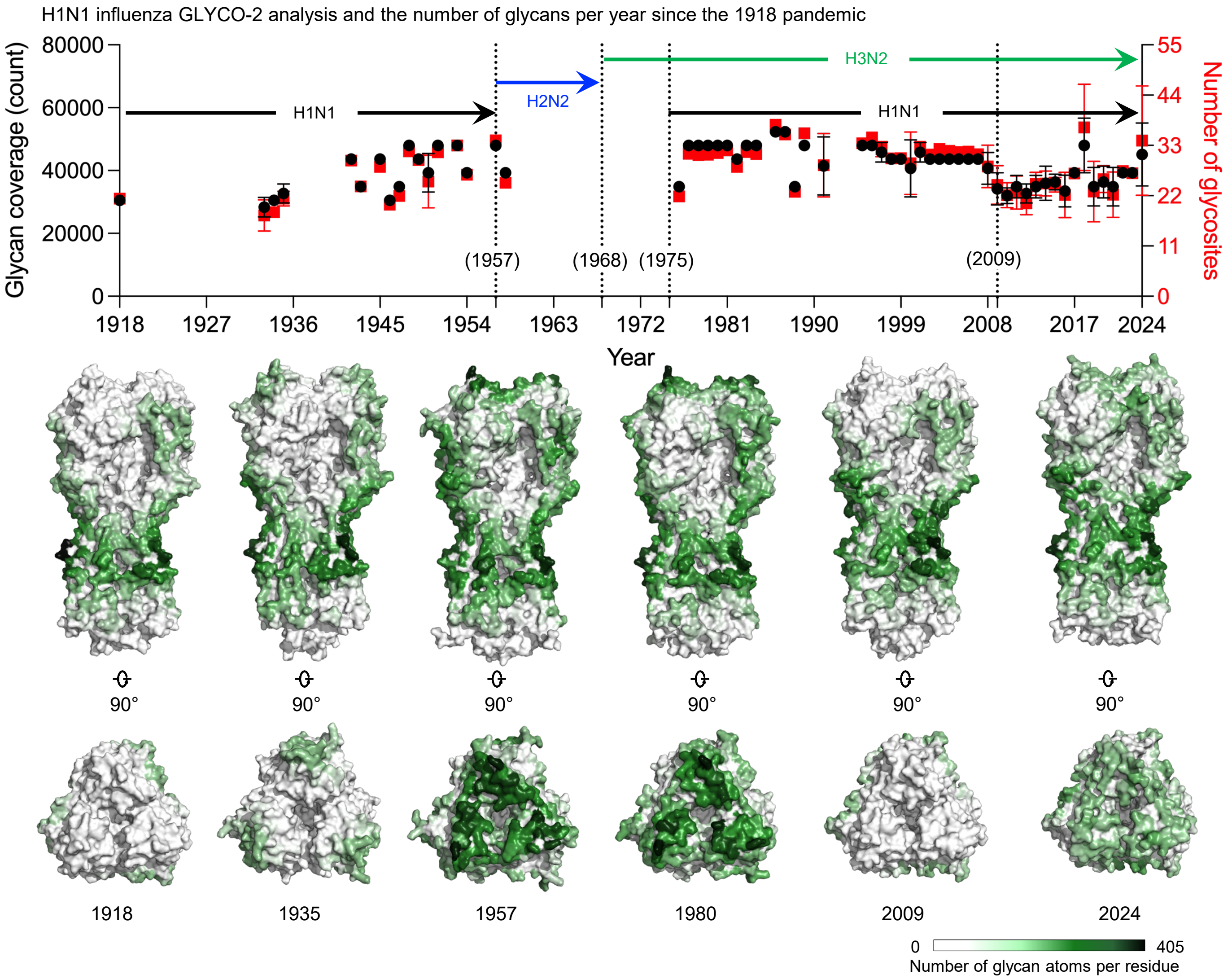

Figure S1. Benchmark study of GLYCO-2 to analyze glycan density of Influenza A H1N1.
Glycan coverage (black) and sequon (red) analysis for Influenza A H1N1 from 1918 to 2024. Corresponding glycan coverage overlaid structures are shown below the plot. A glycan distance cutoff of 23 Å, a surface area of 30 Å², and a cylinder radius of 1.4 Å were used for the calculations. Pandemic periods of H3N2, H2N2, and H1N1 are marked with arrows.

Table S1. 20 test cases for comparing GLYCO-1 and GLYCO-2.

	Viral antigen	PDB	Glycan	Glycan distance cutoff	Surface area	Cylinder radius
1	Ebola	One of PDBs from a MD trajectory	*N*-linked mannose-5 (BGLN, BMAN, AMAN)	23	30	1.4
2	Ebola	3S88	*N*-linked mannose-5 (NAG)	10	30	1.4
3	HIV-1 Env trimer	One of PDBs from a MD trajectory	*N*-linked mannose-5 (BGL, BMA, AMA)	23	30	1.4
4	HIV-1 Env trimer	One of PDBs from a MD trajectory	*N*-linked mannose-9 (BGL, BMA, AMA)	26	30	1.4
5	HIV-1 Env trimer	One of PDBs from a MD trajectory	Site specific (AFU, BGL, AMA, BGA, ANE)	26	30	1.4
6	HIV-1 Env trimer	One of PDBs from a MD trajectory	*N*-linked mannose-5 (BGL, BMA, AMA)	23	30	1.4
7	HIV-1 Env trimer	One of PDBs from a MD trajectory	*N*-linked mannose-5 (BGL, BMA, AMA)	23	30	1.4
8	HIV-1 Env monomer	5FYL – monomer	Incomplete glycans in PDB (NAG, BMA, AMA)	10	30	1.4
9	HIV-1 Env monomer	5FYL – antibody bound monomer complex	Incomplete glycans in PDB (NAG, BMA, AMA)	26	30	1.4
10	HIV-1 Env trimer	5FYL – antibody bound trimer complex	Incomplete glycans in PDB (NAG, BMA, AMA)	10	30	1.4
11	HIV-1 Env trimer	One of PDBs from a MD trajectory	*N*-linked mannose-5 (BGA, BGL, BMA, AMA)	23	30	1.4
12	SARS-CoV-2	One of PDBs from a MD trajectory	*N*-linked mannose-5 (BGL, BMA, AMA)	23	30	1.4
13	SARS-CoV-2	One of PDBs from a different MD trajectory	*N*-linked mannose-5 (BGA, BGL, BMA, AMA)	23	30	1.4
14	SIVmac239	One of PDBs from a MD trajectory	*N*-linked mannose-5, O-linked (BGA, BGL, BMA, AMA)	23	30	1.4
15	influenza HA	One of PDBs from a MD trajectory	*N*-linked mannose-5 (BGL, BMA, AMA)	23	30	1.4
16	influenza HA	H3N2 2020 homology model	*N*-linked mannose-5 (ASM)	23	30	1.4
17	Zika	One of PDBs from a MD trajectory	*N*-linked mannose-5 (BGLN, BMAN, AMAN)	23	30	1.4
18	Zika	One of PDBs from a different MD trajectory	*N*-linked mannose-5 (BGLN, BMAN, AMAN)	23	30	1.4
19	Adhesion domain of human CD2	1GYA	*N*-linked mannose-7 (NAG, BMA, MAN)	23	30	1.4
20	VSG3	6ELC	*O*-linked glycan (NAG, GLC)	10	30	1.4
